# Early Low‐Dose Rituximab for Mild‐to‐Moderate Thyroid Eye Disease: A Preliminary Evaluation of Efficacy and Safety

**DOI:** 10.1155/ije/9495296

**Published:** 2026-01-14

**Authors:** Ming Lu, Luqiang Wang, Xuelian Zhang

**Affiliations:** ^1^ Department of Endocrinology, Beijing Tongren Hospital, Capital Medical University, Beijing, China, ccmu.edu.cn; ^2^ Department of Orthopedics, National Cancer Center/National Clinical Research Center for Cancer/Cancer Hospital, Chinese Academy of Medical Sciences and Peking Union Medical College, Beijing, China, cacms.ac.cn

**Keywords:** early low-dose, immunomodulatory therapy, rituximab, thyroid eye disease, thyrotropin receptor antibody

## Abstract

**Background:**

Thyroid eye disease (TED) is an autoimmune inflammatory condition linked to thyroid dysfunction, which can result in disfigurement and potential vision loss. Recently, immunomodulatory therapies such as rituximab have demonstrated potential benefit in managing TED. However, the optimal dosing and timing of rituximab administration remain uncertain.

**Objective:**

This study aimed to assess the efficacy and safety of early, low‐dose rituximab therapy in patients with mild to moderate TED.

**Methods:**

Eight untreated patients with mild to moderate TED were enrolled in the study. Weekly rituximab infusions at a low dose (100 mg weekly for 4 weeks) were administered, followed by a 16‐week follow‐up period. Thyroid function, thyrotropin receptor antibody (TRAb) levels, ophthalmic examinations, and adverse effects were evaluated at each visit. Additionally, orbital MRI, radionuclide orbital imaging, and clinical activity score (CAS) were performed before treatment and at the final follow‐up.

**Results:**

The mean follow‐up duration was 16 weeks for the eight patients. Detailed baseline patient characteristics were recorded. CD3–CD20 levels decreased significantly after rituximab treatment, compared to baseline levels. TRAb levels also showed a statistically significant decline during the treatment period compared to the baseline levels (*p* = 0.012). The CAS showed a significant decrease from baseline to 16 weeks post‐treatment (*p* = 0.027). There were no significant differences in orbital MRI imaging before and after treatment yet. Nuclear medicine orbital imaging assessment indicated an improvement trend in orbital inflammatory activity among patients. Few adverse effects were observed during the therapy.

**Conclusions:**

The preliminary exploratory study suggested that the early low‐dose rituximab treatment (100 mg weekly for 4 weeks) may be associated with immunological changes (TRAb reduction, B‐cell depletion) in patients with early progressive, mild‐to‐moderate TED. These hypothesis‐generating findings require validation in randomized, placebo‐controlled trials with adequate sample sizes and longer follow‐up.

## 1. Introduction

Thyroid eye disease (TED), also known as Graves’ ophthalmopathy or thyroid‐associated ophthalmopathy, is an autoimmune disorder specific to the organ, closely related associated with thyroid conditions [1, 2]. It holds the highest incidence rate among adult orbital diseases. TED is the primary extrathyroidal manifestation of Graves’ disease, characterized by symptoms like eyelid retraction, bulging eyes, diplopia, restrictive eye movement, corneal exposure, and optic nerve damage [3]. These manifestations pose a significant risk to vision and have a profound impact on the individual’s overall quality of life. TED is characterized by inflammation of the orbital connective tissue, extraocular muscles inflammation and fibrosis, and adipogenesis within the orbits. Despite ongoing research, the complete pathophysiology of TED remains incompletely understood, implicating both the humoral and cell‐mediated immune responses [4].

Treatments for severe TED encompass anti‐inflammation medicines and radiotherapy. Surgical orbital decompression, a last resort for vision‐threatening conditions, aimed at alleviating pressure on the eye [5, 6]. Intravenous glucocorticoids (GC) therapy serves as the first‐line treatment target for active and moderate‐to‐severe cases [7]. However, the side effects of GC’s, including ulcers, infections, weight gain, diabetes, and hypertension, restrict their use in numerous patients. Additionally, a portion of patients exhibit resistance to steroid treatment, while some patients experience relapsing once the steroid is withdrawn [8]. In recent years, alternative immunomodulatory agents with anti‐inflammatory and disease‐modifying capabilities have emerged as promising alternatives [9, 10].

Rituximab is a monoclonal antibody drug approved for the treatment of low‐to‐moderate grade malignant non‐Hodgkin’s lymphoma and has also been employed to treat some autoimmune diseases [11]. Due to its unique pharmacological mechanism, its use in TED patients has been initiated in recent years [12–14]. However, the optimal dosing and timing of rituximab administration remains uncertain.

Treatment of TED usually requires a combination of systemic and ocular therapy. Although there are many treatment options available, most are aimed at patients with moderate to severe TED [15]. The selection of appropriate therapy for those with mild to moderate TED conditions remains controversial.

In this study, we aimed to assess the efficacy and safety of initiating low‐dose rituximab therapy early, particularly for patients with early progressive mild to moderate TED.

## 2. Materials and Methods

### 2.1. Patients

Patients were recruited at Beijing Tongren Hospital from 2023 to 2025. The study was approved by the ethics committees of our hospital (Approval Number: TREC2025‐KY116). Written informed consent was all obtained before patients were recruited. The definition of early progressive TED: Temporal criterion: Recent onset or worsening of TED symptoms within 6 months prior to enrollment. Clinical progression (at least one required): New or increasing proptosis; New or worsening diplopia; Progressive periorbital edema, conjunctival injection, or eyelid retraction. Imaging evidence of active inflammation (at least one required): Increased SPECT/CT uptake in extraocular muscles (URP or URA > 1.0 compared to reference tissue). Inclusion criteria were as follows: (1) untreated patients. Exclusion criteria were as follows: (1) vision‐threatening TED; (2) medically unfit to receive RTX (history of pulmonary tuberculosis, positive hepatitis, absolute neutrophil count < 1.5 × 10^9^/L); (3) pregnant or breastfeeding.

Safety monitoring: (1) Pretreatment screening: All patients underwent screening for HBV, HCV, HIV, and tuberculosis before treatment. (2) Follow‐up monitoring: Patients were monitored for infections at each visit through clinical assessment and complete blood count. Immunoglobulin levels were checked at baseline and week 16. (3) Extended monitoring: We are conducting ongoing follow‐up over 16 weeks to monitor late infections.

### 2.2. Study Design

A total of 8 untreated patients were enrolled in the study. All individuals were treated with RTX weekly of 100 mg for 4 weeks, and antiallergy medications were given before administering to mitigate the risk of allergic reactions. Heart rate and blood pressure were continuously monitored throughout the treatment. Patients were assessed comprehensively before treatment. Continuous weekly (at 1, 2, 3, 4 weeks) treatment included laboratory tests, clinical manifestation, and ophthalmic examination weekly. Orbital magnetic resonance imaging (MRI), radionuclide orbital imaging, and clinical activity score (CAS) measurements were performed before and at 16‐week follow‐up visit. Study endpoints: (1) Primary endpoint: Change in thyrotropin receptor antibody (TRAb) levels from baseline to week 16. (2) Secondary endpoints: Change in CAS, B‐cell count (CD3‐20), thyroid function parameters, orbital imaging parameters (SPECT/CT uptake ratios); safety and tolerability.

### 2.3. Clinical Assessment

All data were collected from medical records. The laboratory tests included the thyroid‐stimulating hormone (TSH, mIU/L), total triiodothyronine (TT3, nmol/L), total thyroxine (TT4, nmol/L), free triiodothyronine (FT3, pmol/L), free thyroxine (FT4, pmol/L), TRAb (IU/L), CD3‐CD20 levels, and cytokines. Baseline assessment included medical history and smoking history, and physical examination was conducted by an endocrinologist. Ophthalmic evaluations including proptosis, visual acuity, and intraocular pressure (IOP) were conducted by an experienced ophthalmologist. The CAS consists of seven items: spontaneous retrobulbar pain, pain on attempted eye movements, conjunctival redness, redness of the eyelids, chemosis, swelling of the caruncle, and swelling of the eyelids; the final score is the sum of all items present. CAS was assessed before and at 16 weeks following up by an experienced ophthalmologist. Orbital MRI scans and radionuclide orbital imaging were performed before and at 16 weeks following up. To quantitate the change of ^99mf^TC‐DTPA PECT/CT, the uptake ratio between the posterior orbit and the occipital (URP) and the uptake ratio between the anterior orbit and the occipital (URA) were assessed by nuclear medicine specialists before and after RTX.

### 2.4. Statistical Analysis

Statistical analyses were performed using SPSS software and GraphPad Prism. Baseline characteristics were presented as mean ± standard deviation for quantitative variables and as percentages for categorical variables. Paired comparisons between baseline and post‐treatment were performed using the Wilcoxon signed‐rank test. For multiple cytokine comparisons, we applied the Benjamini–Hochberg procedure to control the false discovery rate (FDR). Effect sizes were expressed as Hodges–Lehmann median differences with 95% confidence intervals. McNemar’s test was used to analyze changes in paired categorical variables (nuclear medicine orbital imaging inflammatory activity) before and after treatment. For all statistical tests, *p* values of less than 0.05 were statistically significant.

## 3. Results

### 3.1. General Indicators

Baseline patient characteristics are presented in Table 1. The study included 8 patients (2 males and 6 females; age range of 31–52 years). Among the participants, 6 patients suffered with hyperthyroidism and were treated with methimazole (MMI) in the past. Two of them (25%) had stopped MMI and did not receive treatment for their thyroid dysfunction. Among the 6 patients (75%) with hyperthyroidism on MMI, 5 patients maintained a stable dose of MMI and normal stable thyroid function during the follow‐up period, while one required an adjustment in MMI dosage due to elevated indicators (Table S1). All the 8 patients developed TED within a year, duration of 2–12 months without previous therapy of TED. Two cases were smokers. Referring to history of disease, 4 patients exhibited impaired glucose tolerance, and none of them used hypoglycemic medication. Additionally, 2 patients combined cardiovascular disease and three experienced dyslipidemia and got hypolipidemic drug treatment. No participant had hypertension, cerebrovascular disease, diabetes mellitus, or tumors (Table 1).

**Table 1:** Demographic and clinical characteristics of TED patients treated with rituximab.

**Characteristic**	**At baseline (*n* = 8)/(median, IQR)**	**After rituximab therapy (*n* = 8)/(median, IQR)**	**p**

Age (years)	39.88 (8.15)		
Gender (male/female) *n* (%)	2 (25%)/6 (75%)		
Duration of TED (months)	3.88 (3.40)		
Hyperthyroidism, *n* (%)	8 (100%)		
Therapy MMI, *n* (%)	6 (75%)		
Previous treatment of TED	0		
Smoking history, *n* (%)	2 (25%)		
Diabetes, *n* (%)	0		
IGT, *n* (%)	4 (50%)		
Antihypertensive therapy, *n* (%)	0		
Cardiac vascular disease, *n* (%)	2 (25%)		
Cerebral vascular disease, *n* (%)	0		
Dyslipidemia, *n* (%)	3 (37.5%)		
Hypolipidemic drug, *n* (%)	3 (37.5%)		
Malignant tumor, *n* (%)	0		

	**(Median, IQR)**	**(Median, IQR)**	**p**

Systolic blood pressure (mmHg)	125.50 (114.50–129.00)	125.00 (121.25–130.50)	0.122
Diastolic blood pressure (mmHg)	79.00 (69.50–87.25)	79.50 (75.25–80.25)	1.000
FPG (mmol/L)	5.00 (4.65–5.40)	4.80 (4.45–4.80)	0.269
HPG (mmol/L)	123.00 (119.50–142.00)	127.00 (123.50–141.00)	0.307
HbA1c (%)	5.30 (5.05–5.65)	5.20 (5.10–5.50)	0.734
Triglycerides (mmol/L)	1.03 (0.94–1.80)	1.00 (0.68–1.18)	0.310
Total cholesterol (mmol/L)	4.36 (3.85–5.25)	4.00 (3.63–4.25)	0.128
HDL‐cholesterol (mmol/L)	1.21 (1.05–1.40)	1.16 (1.11–1.23)	0.499
LDL‐cholesterol (mmol/L)	2.73 (2.35–3.20)	2.59 (2.45–2.80)	0.499
Alanine aminotransferase (U/L)	18.00 (12.00–23.00)	14.00 (12.50–28.50)	0.932
Creatinine (mmol/L)	52.00 (46.50–66.50)	59.00 (55.00–70.00)	0.176
Urine acid (mmol/L)	343.00 (319.50–362.00)	323.00 (287.00–397.00)	0.866
FINS (mU/L)	10.90 (6.94–14.40)	10.00 (8.00–14.00)	0.735
HOMA‐IR	2.02 (1.54–3.32)	2.13 (1.48–2.53)	0.499
Cortisol (μg/dL)	12.27 (9.98–18.27)	8.17 (5.88–11.50)	**0.043**
ACTH (pg/mL)	31.20 (30.50–72.40)	29.80 (21.40–45.85)	0.128

*Note:* Clinical characteristics were presented with median, IQR for quantitative variables and as percentages for categorical variables. HPG, Hemoglobin; HbA1c, Hemoglobin A1C; FINS, Fasting Insulin; HOMA‐IR; Homeostasis Model Assessment‐Insulin Resistance Index. Bold values indicate statistically significant differences with *p* < 0.05.

Abbreviations: FPG, Fasting Plasma Glucose; IGT, Impaired Glucose Tolerance.

Compared to baseline, there were no significant changes in the blood pressure following rituximab treatment, with all patients maintaining normal blood pressure level. There was no significant difference in fasting and postprandial blood glucose, as well as hemoglobin before and after treatment. There was no significant difference in lipid profiles before and after treatment, and no significant differences in liver and kidney functions. There was no significant difference in insulin resistance index. Notably, cortisol levels decreased significantly after treatment compared to baseline (*p* = 0.043), while adrenocorticotropic hormone (ACTH) levels decreased, though this difference did not reach statistical significance (Table 1). These indicators showed stable basic characteristics before and after rituximab treatment.

### 3.2. Effects of RTX Treatment on CD20 and Cytokines

In assessing cellular inflammatory factors, it was found that there was no significant difference in levels of interferon, complement, and TNF‐α. Given rituximab targeting and removal of B‐cell tumors in vivo, testing for CD20 expression was performed prior to rituximab administration. In this study, CD3–CD20 levels significantly decreased after rituximab treatment, showing marked differences compared to baseline (*p* = 0.018). After FDR adjustment, no cytokine reached statistical significance, indicating exploratory trends rather than a definitive cytokine response. Additionally, IgM levels significantly declined after treatment (*p* = 0.043), while other immunoglobulin levels showed no significant change before or after treatment. These indicated that rituximab might influence the immunity markers in whole body (Table 2).

**Table 2:** Changes of B and T cell subsets, cytokines, and immunoglobulins in TED patients after rituximab treatment.

Characteristic	At baseline (median, IQR)	After rituximab therapy (median, IQR)	*p*
CD3‐CD20	11.33 (3.85–19.05)	0.03 (0.00–0.05)	**0.018**
IL‐1β (pg/mL)	3.50 (2.17–4.49)	5.59 (4.48–9.90)	0.144
IL‐2 (pg/mL)	1.69 (1.63–1.78)	0.25 (0.00–0.76)	0.068
IL‐4 (pg/mL)	1.98 (1.70–2.00)	1.75 (1.42–2.31)	0.715
IL‐5 (pg/mL)	2.76 (2.59–3.15)	2.79 (1.89–3.75)	0.715
IL‐6 (pg/mL)	2.37 (1.92–3.44)	1.82 (1.70–2.32)	0.273
IL‐8 (pg/mL)	2.36 (1.79–2.71)	3.65 (2.70–5.22)	0.273
IL‐10 (pg/mL)	1.35 (0.93–1.72)	0.88 (0.83–0.95)	0.068
IL‐12p70 (pg/mL)	1.69 (1.48–1.97)	1.34 (1.23–1.48)	0.068
IL‐17 (pg/mL)	2.10 (1.54–2.39)	1.85 (1.58–2.22)	1.000
TNF‐α (pg/mL)	2.52 (1.35–3.45)	1.18 (0.56–2.79)	0.715
INF‐α (pg/mL)	1.36 (1.08–1.52)	0.88 (0.45–1.27)	0.715
INF‐β (pg/mL)	15.62 (12.63–16.18)	19.16 (16.32–30.94)	0.144
IgG (g/L)	12.00 (10.00–12.00)	11.00 (10.00–13.00)	0.317
IgA (g/L)	1.61 (1.56–1.81)	1.79 (1.52–2.16)	0.353
IgM (g/L)	1.24 (1.18–1.29)	1.20 (0.94–1.28)	**0.043**
C3 (mg/L)	1121.19 (1059.08–1239.35)	1092.00 (1012.50–1168.13)	0.500
C4 (mg/L)	249.00 (227.10–322.40)	231.50 (218.00–341.80)	0.893
C1q (mg/L)	212.00 (191.00–222.00)	195.00 (180.00–199.00)	0.138

*Note:* Cytokines were expressed as median, IQR. IL, Interleukin; INF, Interferon; Ig, Immunoglobulin; C, Complement. Wilcoxon signed‐rank tests were utilized to compare between two groups. Bold font indicated a statistically significant difference with *p* < 0.05.

Abbreviation: TNF, Tumor Necrosis Factor.

### 3.3. Effects of RTX Treatment on Thyroid Function, TRAb, and CAS

We evaluated various clinical indicators in the study at baseline, during the 2nd, 3rd, 4th, and 16th weeks post‐treatment follow‐up. Changes in the levels of thyroid hormones levels were observed from the onset of treatment to 16‐week follow‐up. There was no significant difference in the levels of FT3 and FT4, and the level of TSH showed significant improvement compared to the baseline (Wilcoxon *p* = 0.043). The primary endpoint, TRAb levels significantly diminished from 4.79 (IQR: 2.58–14.23) IU/L at baseline to 1.99 (IQR: 1.31–5.33) IU/L at 16 weeks, (Hodges–Lehmann estimate: −2.81, 95% CI: −10.8–−0.87, Wilcoxon *p* = 0.012) (Table 3, Figure 1(a)). CAS also exhibited a significant reduction from 1.5 (IQR: 1.0–2.0) at baseline to 1.0 (IQR: 0.0–1.0) in 16 weeks after treatment (Hodges–Lehmann estimate: −1.0, 95% CI: −1.0–−0.0, Wilcoxon *p* = 0.027) (Table 3, Figure 1(b)). Individual TRAB and CAS values for all patients are shown in Table S1. There was no significant change in IOP before or after treatment and no significant decrease in visual acuity (Table 3).

**Table 3:** Changes of clinical and laboratory parameters in TED patients after rituximab treatment.

Characteristic	At baseline (median, IQR)	After rituximab therapy (median, IQR)	Median diff (HL, 95% CI)	*p*
TT3 (nmol/L)	1.69 (1.38–1.85)	1.45 (1.42–1.56)	−0.27 (−0.66–0.27)	0.271
TT4 (nmol/L)	114.80 (103.05–126.95)	109.60 (107.65–113.85)	−4.90 (−24.00–13.60)	0.612
FT3 (pmol/L)	5.41 (4.67–5.87)	4.76 (4.60–5.02)	−0.20 (−0.95–0.04)	0.128
FT4 (pmol/L)	11.15 (10.50–13.07)	9.51 (8.28–10.83)	−2.30 (−6.66–0.37)	0.128
TSH (mIU/L)	0.07 (0.01–0.27)	1.48 (0.65–3.82)	1.41 (0.01–3.72)	**0.043**
TRAb (IU/L)	4.79 (2.58–14.23)	1.99 (1.31–5.33)	−2.81 (−10.80–−0.87)	**0.012**
CAS	1.5 (1.0–2.0)	1.0 (0.0–1.0)	−1.0 (−1.0–0.0)	**0.027**
IOP (OD)	14.90 (14.20–18.75)	13.50 (12.70–18.50)	−1.50 (−3.10–0.50)	0.113
IOP (OS)	16.00 (13.23–17.50)	13.50 (12.32–16.25)	−0.60 (−3.00–1.00)	0.340
Vision (L)	1.00 (1.00–1.00)	1.00 (1.00–1.00)	0.00 (0.00–0.00)	0.914
Vision (R)	1.00 (1.00–1.00)	1.00 (1.00–1.05)	0.00 (0.00–0.10)	0.633

*Note:* Laboratory parameters were expressed as median, IQR. IOP, Intraocular Pressure. Wilcoxon signed‐rank tests were used to compare in baseline and 16 weeks post‐rituximab treatment groups. Bold font indicated a statistically significant difference with *p* < 0.05.

Abbreviations: CAS, Clinical Activity Score; OD, Oculus Dexter; OS, Oculus Sinister.

Figure 1TRAb levels and CAS during the treatment of rituximab. (a) Changes of TRAB levels during the treatment of rituximab. (b) Wilcoxon signed‐rank test was performed to compare CAS in baseline and after rituximab therapy.

**Figure:**
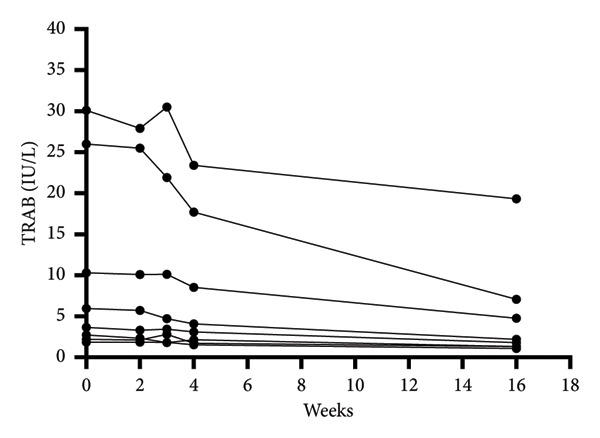
(a)

**Figure:**
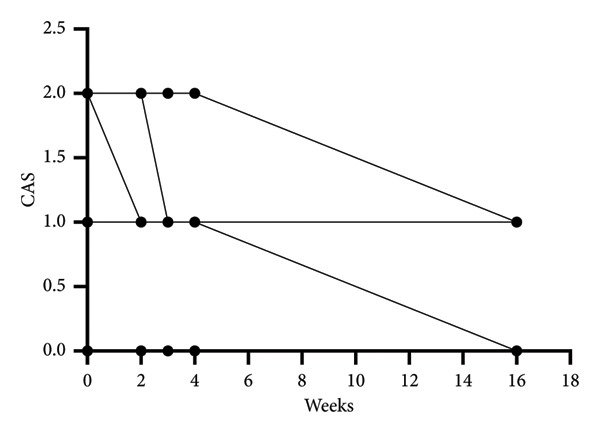
(b)

### 3.4. Effects of RTX Treatment on Imaging Parameters

**Figure 2:**
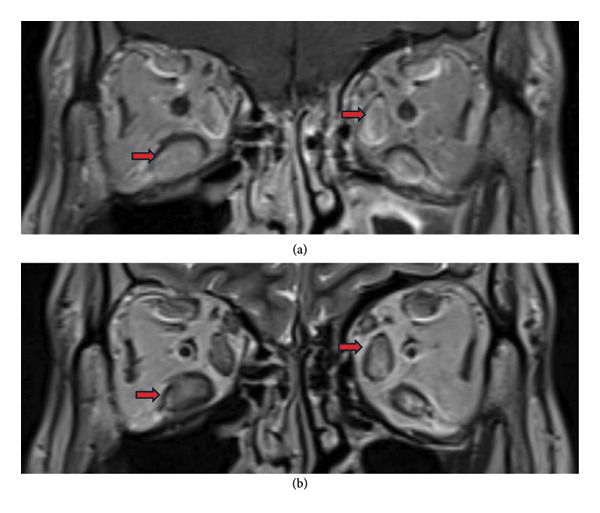
Pre‐ and post‐treatment orbital MRI imaging. Pre‐ and post‐treatment orbital MRI imaging showing extraocular muscle involvement. (a) Representative T2‐weighted coronal MRI images before treatment showing bilateral inferior rectus muscle thickening and enhancement (arrows). (b) Post‐treatment images at 16 weeks showing stable muscle involvement without significant morphological changes. While quantitative differences were not statistically significant due to short follow‐up, the images demonstrate that no progression of muscle fibrosis occurred during treatment. Inner rectus and inferior rectus muscles are the most affected muscles in TED and were marked with red arrows.

**Figure 3:**
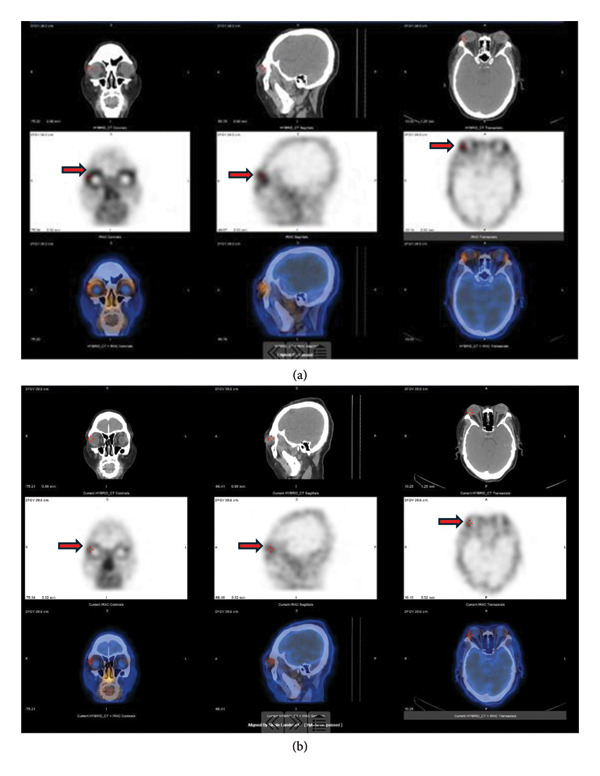
Pre‐ and post‐treatment nuclear medicine orbital imaging. Pre‐ and post‐treatment nuclear medicine orbital imaging (99mTc‐DTPA SPECT/CT). (a) Pretreatment images showing moderate to high uptake in orbital tissues indicating inflammation (arrows indicate areas of increased tracer uptake). (b) Post‐treatment images at 16 weeks showing reduced uptake, consistent with decreased inflammatory activity. Five patients (62.5%) showed improvement from moderate to mild inflammatory activity.

To comprehensively assess the overall outcome of patients with TED, dynamic orbital imaging was conducted. Before and after rituximab treatment, unilateral ocular muscle involvement was found in 62.5%, bilateral ocular muscle involvement in 25%, and lacrimal gland prolapse in 75%. Due to the short follow‐up period, there were no significant differences in orbital imaging before and after treatment yet (Table 4, Figure 2). Orbital imaging of nuclear medicine was employed to monitor the alterations in inflammatory activity. The results revealed that after treatment with rituximab, the inflammatory activity of orbital lesions changed from moderate to mild in 5 patients (62.5%) with TED, while 3 patients (37.5%) maintained mild inflammation throughout the study period. At the end of the follow‐up period, all patients exhibited mild inflammation on nuclear medicine orbital imaging, and no patients experienced worsening inflammatory activity (McNemar’s exact test, *p* = 0.063). URP exhibited a reduction from 1.083 (0.892–1.290) to baseline to 0.972 (0.796–1.262) (Wilcoxon *p* = 0.179) in 16 weeks after treatment. URA also showed a decrease from 1.344 (1.208–1.472) in baseline to 1.265 (1.130–1.395) (Wilcoxon *p* = 0.134) in 16 weeks after treatment. Individual URP and URA for all patients are shown in Table S1. The URP and URA showed a decrease trend after treatment, suggesting the potential clinical improvement in orbital inflammatory activity in patients with TED. These findings represent preliminary trends (Table 4, Figure 3).

**Table 4:** Evaluation of therapeutic effects of TED patients after rituximab treatment.

Characteristic	At baseline (*n*, %)/(median, IQR)	After rituximab therapy (*n*, %)/(median, IQR)	*p* value
Diplopia	0	0	
Orbital MRI			
unilateral ocular muscle thickening	5 (62.5%)	5 (62.5%)	
lateral ocular muscle thickening	2 (25%)	2 (25%)	
lacrimal glands prolapse	6 (75%)	6 (75%)	
Orbital Imaging of nuclear medicine			
mild	3 (37.5%)	8 (100%)	0.063
moderate	5 (62.5%)	0 (0%)	
severe	0	0	
URP	1.083 (0.892–1.290)	0.972 (0.796–1.262)	0.179
URA	1.344 (1.208–1.472)	1.265 (1.130–1.395)	0.134

*Note:* Evaluations of therapeutic efficacy of TED were expressed as percentages relative to the total cohort. All the detailed characteristics are listed in Table 4. URP: an uptake ratio between the posterior orbit and the occipital region. URA: an uptake ratio between the anterior orbit and the occipital region. *t*‐tests were used to compare URP and URA in baseline and 16 weeks post‐rituximab treatment groups. McNemar’s test for paired categorical data.

### 3.5. Side Effects

Throughout the treatment, we closely monitored all endpoint events focusing on rituximab‐related adverse reactions, including viral hepatitis, severe mucosal reactions and some other diarrheal and gastrointestinal reactions at 16‐week follow‐up. Only one case of urticaria was documented, and no other serious adverse reactions were noted. Overall, the safety of rituximab was confirmed during the treatment period.

## 4. Discussion

TED is a refractory disease with several therapeutic options available, including GC, immunological agents, biologics, orbital linear accelerators, and additional treatments. Despite a range of therapeutic approaches mentioned in the literature and relevant guidelines [16], the therapeutic effects vary greatly due to individual differences. Rituximab, by depleting CD19+ and CD20+ B cells, has been utilized to treat B‐cell lymphomas since 1997 and is also effective for treating rheumatoid arthritis and membranous nephropathy [17–19]. It was also introduced for managing active TED in 2006 [20]. And subsequent nonrandomized controlled studies have further supported its use to treat GC‐resistant TED cases.

The patients in this study were middle‐aged individuals with a recent diagnosis of the first episode TED. All patients had a history of hyperthyroidism, were positive for TRAb, and most were treated with MMI. All patients had their thyroid function at baseline checked. Decrease in TRAb was observed during short‐term treatment with rituximab, which might augment by antithyroid drugs as well as rituximab, which was consistent with previous research findings. In our study, most patients maintained stable MMI dosing with controlled thyroid function, and patient with dose reduction did not drive the association between medication changes and outcomes. MMI variation is unlikely to have confounded the observed treatment effect of rituximab. The exact mechanism of action of rituximab on TRAb needs to be further explored.

Inflammatory factor CD20 was decreased during rituximab treatment, highlighting a unique mechanism of action of rituximab itself. Immunoglobulin tests suggested modulation of the immune system by rituximab. In addition, from baseline to the end of follow‐up, CAS and nuclear medicine orbital imaging demonstrated that patients might achieve improvement in inflammatory response with 4 weeks of rituximab treatment. Due to the short time frame, no exacerbations, optic nerve compression, or other vision‐threatening conditions were observed on orbital imaging in any of the patients. These findings suggest potential therapeutic efficacy.

There is still controversy about the population, indications, and range of doses in using rituximab [21]. Multiple studies have explored the therapeutic options of rituximab in patients with TED. Early studies favored high doses (500 or 1000 mg, or 375 mg/m^2^ weekly for 4 weeks), but these regimens were associated with side effects such as fever, infusion reactions, abdominal pain, and joint pain [20]. After that, some studies had attempted to use lower‐dose rituximab for treatment. One study reported that 100–400 mg of low‐dose rituximab achieved clinical improvement in 87% of TED patients after 2 months [22]. Another study demonstrated the efficacy of 100 mg rituximab combined with GC or other immunosuppressants in TED patients [23]. A further investigation confirmed that a single 100 mg dose of rituximab effectively treated active moderate‐to‐severe TED [24]. A smaller dose of rituximab (125 mg/m^2^ body surface area) also showed effectiveness in managing active moderate‐to‐severe TED [25]. Overall, due to the lack of randomized trials, the appropriate dosage of rituximab has not been fully elucidated. Our findings suggested that low‐dose rituximab can benefit untreated TED patients.

In addition, the optimal timing for rituximab treatment remains uncertain. Two landmark randomized controlled trials have provided contrasting results regarding rituximab efficacy in TED. In one study, Stan and Salvi reported no significant difference in the effectiveness versus placebo with an average course of exophthalmos of about 12 months [26], while in another study, Salvi et al. demonstrated that the efficacy of rituximab treatment was significantly better than methylprednisolone treatment, with subjects suffering a duration of exophthalmos of 4.6 months [27]. A subsequent meta‐analysis by Shen et al. concluded that rituximab may be effective for TED treatment, though the evidence quality was limited by study heterogeneity and methodological differences [28]. To some extent, the efficacy of rituximab in TED might depend on the course and progression of the disease. Our study suggests that early initiation of rituximab can be beneficial.

Unlike previous studies, the treatment regimen of rituximab in the present study was characterized by a small dose and short course of treatment. The present study reported a 4‐week, low‐dose rituximab regimen, which would be easier, safer, and faster compared to high‐dose rituximab (500 mg), high‐dose, long‐duration (4–6 months) talizumab, and the classic long‐duration (12 weeks), high‐dose intravenous GC therapy.

TRAb itself is a contributing factor in thyroid‐related eye disease [29], and TRAb levels may influence the progression of TED to some extent [30]. In these cases, applying rituximab was effective for treating TED patients while reducing TRAb levels. The efficacy and safety of this regimen are supported by the significant decrease in CAS, the improvement in nuclear medicine orbital imaging, and the patients’ complaints. There are many choices of TED treatment regimens, and although the current guidelines do not yet recommend the use of rituximab in patients with mild‐to‐moderate TED, the results of our study explored the use of a short course of low‐dose rituximab treatment in patients with first‐episode, mild‐to‐moderate TED who combined hyperthyroidism. This treatment option might be a novel option.

It should be noted that there are some contraindications to rituximab therapy [31]. Patients who are allergic to the components of the drug, with severe active infections or severely compromised immune response, with severe heart failure, should avoid using this drug. During treatment, attention should be paid to possible adverse reactions such as systemic reactions, skin reactions, and immune system reactions. In this study, the safety of rituximab treatment was closely monitored. The incidence of adverse reactions was found to be lower when using the regimen of rituximab (100 mg weekly for 4 weeks) compared to other biologics, as measured by patient complaints, physical signs, blood analysis, liver and renal function, lung imaging, and other relevant indicators.

Some of the mild TED patients are self‐limiting, but many of them progress to moderate‐to‐severe TED, which requires active pharmacological intervention. Early intervention can minimize or delay the progression of moderate‐to‐severe TED and reduce the risk of blindness in this population. Our study focused on a distinct patient population: early progressive TED demonstrable inflammation on functional imaging. Emerging evidence suggests that functional imaging, such as SPECT/CT, may detect inflammation earlier than clinical signs. Patients with progressive symptoms and imaging‐confirmed inflammation may represent a therapeutic window for early intervention. For patients with first‐episode, mild‐to‐moderate TED, whether to treat them and whether to use aggressive or standard treatment remain to be explored. In this study, we adopted an early intervention programmed for the above patients. We found that the patients’ hyperthyroidism as well as ocular signs improved, which would help to reduce the severe TED risk. During the TED treatment process, we first need to control the thyroid function, aiming for rapid normalization. On this basis, the prognosis of patients with mild‐to‐moderate TED could be improved through the application of low‐dose, short‐course rituximab therapy, providing a potential novel therapeutic option.

There are some limitations to our study. The absence of a control group is a significant and fundamental limitation of our study, preventing definitive causal attribution of observed improvements to rituximab treatment versus the natural disease course. We cannot exclude spontaneous improvement. The natural history of TED is variable, and spontaneous improvement can occur, particularly in mild cases. In a recent study published in JAMA Ophthalmology in 2025 [32], change in CAS and autoimmune thyroid antibodies showed no difference in control group at 3 months. In another study [33], there was no significant decrease in TRAB levels and CAS after 3 months of intervention in placebo group. In our study, we showed a significant decrease in TRAB levels in rituximab, to some extent, reflecting the effectiveness. Compared with natural history data from placebo arms of published RCTs, it suggests our TRAb reduction exceeds typical spontaneous changes. This study serves as a preliminary and hypothesis‐generating investigation. Future randomized controlled trials with appropriate control groups are essential to establish the true efficacy of early low‐dose rituximab in mild to moderate TED. No formal sample size or power calculation was performed a priori, as this was designed as an exploratory pilot study. Some potential biases have been shown in our study. Our small, nonrandomized sample may not be representative of the broader TED population. The single‐center design may limit generalizability. While TRAb and CAS are relatively objective measures, some components of clinical assessment may be subject to observer bias. The 16‐week follow‐up is another limitation, insufficient to assess long‐term clinical outcomes, disease relapses, or delayed safety events. Structural changes on MRI typically require 6–12 months to manifest. Clinical improvements may continue to evolve for 6–12 months after treatment completion. The study excluded patients with significant comorbidities such as vision‐threatening TED. These exclusions limit generalizability to broader TED populations. Due to the limited number of cases enrolled in this study, our research and results need to be confirmed and extended to a larger and more diverse population and a larger age range with longer follow‐up. The study enrolled a population with fewer comorbidities, and whether it can be used in a population with more comorbidities needs to be further investigated.

Our findings suggest potential benefits of early low‐dose rituximab in mild to moderate TED, definitive evidence requires validation through adequately powered randomized controlled trials. Given the small sample size, lack of control group, and short follow‐up period, these findings should be considered hypothesis‐generating. Future studies should include appropriate control groups, longer follow‐up periods, and larger sample sizes to establish the true efficacy and optimal timing of rituximab therapy in this patient population.

## Conflicts of Interest

The authors declare no conflicts of interest.

## Author Contributions

Ming Lu collected and analyzed the data and wrote the manuscript. Luqiang Wang helped to analyze the data and gave the suggestions. Xuelian Zhang designed the idea and wrote and edited the manuscript.

## Funding

This work was supported by grants from the National Natural Science Foundation of China (82100829, 81300726) and the Beijing Municipal Science and Technology Commision (Z151100004015021).

## Supporting Information

Individual clinical characteristics, such as MMI treatment details, individual outcome measures, and URP and URA for all patients are listed and shown in supporting table.

Table S1: Individual patient data showing baseline characteristics, concomitant medication, and key outcome measures before and after rituximab treatment. Abbreviations: MMI, methimazole; No, indicates no antithyroid medication; TRAb, thyroid receptor antibody; CAS, Clinical Activity Score; URP: an uptake ratio between the posterior orbit and the occipital region. URA: an uptake ratio between the anterior orbit and the occipital region; R, right eye; L, left eye; Both right (R) and left (L) eye measurements are provided.

## Supporting information


**Supporting Information** Additional supporting information can be found online in the Supporting Information section.

## Data Availability

The data that support the findings of this study are available from the corresponding author upon reasonable request.
