# Supplementary material for: Early Low‐Dose Rituximab for Mild‐to‐Moderate Thyroid Eye Disease: A Preliminary Evaluation of Efficacy and Safety
Source: Int J Endocrinol. 2026 Jan 14;2026:9495296. doi: 10.1155/ije/9495296 (PMC12800737; doi:10.1155/ije/9495296)
Supplement: Supplementary file 1 — Supporting Information Additional supporting information can be found online in the Supporting Information section. [file IJE-2026-9495296-s001.docx]

**Table S1** Individual Patient Clinical Characteristics, Treatment Details, and Outcome Measures after rituximab treatment

| data | At baseline | After Rituximab | At baseline | After Rituximab | At baseline  CAS | After Rituximab | At  baseline | After Rituximab | At  baseline | After Rituximab |
| --- | --- | --- | --- | --- | --- | --- | --- | --- | --- | --- |
| Patient No. | Drugs  treatment | Drugs  treatment | TRAB  (IU/L) | TRAB  (IU/L) |  | CAS | PECT/CT uptake URP | PECT/CT uptake URP | PECT/CT  uptake URA | PECT/CT uptake URA |
|  |  |  |  |  |  |  | R, L | R, L | R, L | R, L |
| No.1 | MMI 10mg | MMI 10mg | 30.10 | 19.30 | 2.0 | 1.0 | 1.48,1.48 | 1.36,1.27 | 2.61,2.40 | 1.73,1.67 |
| No.2 | MMI 5mg | MMI 5mg | 10.30 | 4.75 | 2.0 | 0.0 | 1.10,0.87 | 0.79,0.67 | 1.60,1.20 | 1.14,1.19 |
| No.3 | No | No | 2.72 | 1.30 | 1.0 | 0.0 | 1.06,1.01 | 0.87,1.01 | 1.43,1.37 | 1.36,1.37 |
| No.4 | MMI 10mg | MMI 10mg | 5.95 | 2.18 | 1.0 | 1.0 | 0.88,0.87 | 0.61,0.58 | 1.30,1.30 | 1.07,0.96 |
| No.5 | MMI 2.5mg | MMI 2.5mg | 1.83 | 1.07 | 0.0 | 0.0 | 0.81,0.90 | 1.08,1.26 | 1.21,1.09 | 1.27,1.26 |
| No.6 | MMI 20mg | MMI 10mg | 26.01 | 7.07 | 2.0 | 1.0 | 1.35,1.03 | 0.81,0.90 | 1.62,1.36 | 1.10,1.11 |
| No.7 | No | No | 3.64 | 1.80 | 1.0 | 1.0 | 1.12,1.12 | 0.94,1.12 | 1.35,1.33 | 1.46,1.46 |
| No.8 | MMI 5mg | MMI 5mg | 2.18 | 1.31 | 2.0 | 1.0 | 1.36,1.27 | 1.50,1.59 | 1.06,1.15 | 1.27,1.26 |
